# Plasma Circulating Metabolites Associated With Steatotic Liver Disease and Liver Enzymes: A Multiplatform Population-Based Study

**DOI:** 10.1016/j.gastha.2024.09.006

**Published:** 2024-09-12

**Authors:** Yasir J. Abozaid, Ibrahim Ayada, Laurens A. van Kleef, Neil J. Goulding, Jessica S. Williams-Nguyen, Robert C. Kaplan, Robert J. de Knegt, Lynne E. Wagenknecht, Nicholette D. Palmer, Nicholas J. Timpson, Jill M. Norris, Yii-Der Ida Chen, M. Arfan Ikram, Willem Pieter Brouwer, Mohsen Ghanbari

**Affiliations:** 1 Department of Epidemiology, Erasmus MC, University Medical Center Rotterdam, Rotterdam, The Netherlands; 2 Department of Gastroenterology and Hepatology, Erasmus MC, University Medical Center Rotterdam, Rotterdam, The Netherlands; 3 Medical Research Council (MRC) Integrative Epidemiology Unit, University of Bristol, Bristol, UK; 4 Population Health Sciences, Bristol Medical School, University of Bristol, Bristol, UK; 5 Department of Medical Education and Clinical Sciences, Washington State University, Elson S. Floyd College of Medicine, Seattle, Washington; 6 Department of Epidemiology and Population Health, Albert Einstein College of Medicine, Bronx, New York; 7 Department of Public Health Sciences, Wake Forest School of Medicine, Winston-Salem, North Carolina; 8 Department of Biochemistry, Wake Forest School of Medicine, Winston-Salem, North Carolina; 9 Colorado School of Public Health, University of Colorado Anschutz Medical Campus, Aurora, Colorado; 10 The Institute for Translational Genomics and Population Sciences, Department of Pediatrics, The Lundquist Institute for Biomedical Innovation at Harbor-UCLA Medical Center, Torrance, California

**Keywords:** Metabolomics, SLD, Liver Enzymes, General Population, Plasma Metabolites

## Abstract

**Background and Aims:**

Steatotic liver disease (SLD) is the most common chronic liver disease strongly associated with metabolic dysfunction, but its pathogenesis remains incompletely understood. Exploring plasma circulating metabolites may help in elucidating underlying mechanisms and identifying new biomarkers for SLD.

**Methods:**

We examined cross-sectionally the association between plasma metabolites and SLD as well as liver enzymes using data from 4 population-based cohort studies (Rotterdam study, Avon Longitudinal Study of Parents and Children, The Insulin Resistance Atherosclerosis Family Study, and Study of Latinos). Metabolites were assessed in the Nightingale platform (n = 225 metabolites) by nuclear magnetic resonance spectroscopy and in the Metabolon platform (n = 991 metabolites) by ultra-high-performance liquid chromatography-mass spectrometry. Serum levels of liver enzymes (alanine aminotransferase, aspartate aminotransferase, and gamma-glutamyl transpeptidase) were measured and SLD was diagnosed by ultrasound or computed tomography scan. Logistic and linear regression models were performed per cohort and meta-analyzed. A false discovery rate < 0.05 was considered as significant threshold.

**Results:**

Several metabolites were significantly associated with SLD and liver enzymes, of which 21 metabolites were associated with both traits. The most significant associations were observed with phenylalanine, triglycerides in (high-density lipoprotein, intermediate-density lipoprotein, and small low-density lipoprotein), fatty acid (FA) ratios of (18:2 linoleic acid-to-total FA, omega 6 FA-to-total FA, and polyunsaturated FA-to-total FA) from the Nightingale and glutamate and sphingomyelin from the Metabolon platform. Other associated metabolites were mainly involved in lipid, amino acid, carbohydrates, and peptide metabolism.

**Conclusion:**

Our study indicates a landscape of circulating metabolites associated with SLD. The identified metabolites may contribute to a better understanding of the metabolic pathways underlying SLD and hold promising for potential biomarkers in early diagnosis and monitoring of the disease.

## Introduction

Steatotic liver disease (SLD) is the most common chronic liver disease, affecting over 30% of the global population, and generally represents a hepatic manifestation of the metabolic syndrome.^1^^,^^2^ SLD, and specifically metabolic dysfunction–associated fatty liver disease can progress to metabolic dysfunction–associated steatohepatitis (MASH) in approximately 20%–25% of patients.^3^ This progression may lead to liver cirrhosis, development of hepatocellular carcinoma, and ultimately liver failure, necessitating liver transplantation. Among patients listed for liver transplantation, metabolic dysfunction–associated fatty liver disease specifically MASH, is currently one of the most rapidly increasing indications for transplantation.^4^

SLD is characterized by the accumulation of lipids, mainly triacylglycerol, in hepatocytes.^5^ Hepatic lipid metabolism is regulated by a combination of the uptake and export of fatty acids (FAs), de novo lipogenesis, and fat utilization by β-oxidation.^6^ When there is a disruption in the balance between these pathways, it can result in the accumulation of hepatic lipids, leading to MASH reflected by elevated liver enzymes.^7^ This disruption also triggers prolonged activation of inflammatory and fibrotic pathways, potentially advancing to more severe stages of liver disease. Currently, evidence shows that SLD is strongly associated with metabolic risk factors such as obesity, insulin resistance and dyslipidemia.^8^^,^^9^ The molecular mechanisms underlying SLD pathophysiology are complex, multifactorial, and still incompletely understood. Consequently, therapeutic options are primarily limited to lifestyle interventions.^10^^,^^11^ Nevertheless, numerous novel compounds for the treatment of MASH are currently under investigation. Recently, the US Food and Drug Administration approved resmetirom as the first drug for MASH.^12^

Given the lack of effective therapeutic options and the progressive detrimental natural course of SLD, it is of great importance to diagnose the disease at an early stage. Although several noninvasive tests are currently available to aid in the diagnosis of SLD, the gold standard for the diagnosis remains liver biopsy, which is hampered by its invasive nature.^13^^,^^14^ Moreover, important knowledge gaps remain with regard to the disease pathophysiology. Therefore, there is a great need to further unravel important underlying pathways which could help to the identification of noninvasive biomarkers for early detection and classification of SLD and may reveal new avenues for therapeutic development.

Recent metabolomics studies have shown promise in identifying metabolic signatures that may reveal the pathological underpinnings of complex diseases (ie, the major impact of genetic variations and metabolic alterations), including SLD.^15^^,^^16^ These studies have demonstrated several potential pathways which might contribute to the development of SLD, yet the majority of them were limited by either the small number of cases (typically<50) ^17^ or the number of metabolites investigated (typically 100–200).^18^ To date, only a few of these metabolites have been validated at the population level and even very few have been suggested as biomarkers to be used in clinical practice.^19^^,^^20^

Hence, this study aimed to investigate the cross-sectional association between an extensive array of circulating metabolites and SLD, as well as liver enzymes, using data from population-based cohort studies, which may help to elucidate the pathophysiological mechanisms of SLD and also for identification of potential biomarkers for early diagnosis.

## Methods

### Study Populations

#### Rotterdam study (RS)

The Rotterdam Study (RS) currently consists of 17,931 individuals aged 40 years or older from the Ommoord region of Rotterdam. It is a population-based study that comprises 4 distinct cohorts. The objectives and design of the RS have been published elsewhere.^21^ The study includes multiple follow-up visits for each cohort. At each visit, participants completed questionnaires, underwent physical examinations, and provided fasting blood samples. Further details of the RS and each other cohort are thoroughly explained in Supplementary data.

#### Avon Longitudinal Study of Parents and Children (ALSPAC)

The study invited pregnant women residing in Avon, UK, with expected delivery dates between April 1, 1991, and December 31, 1992, to participate. A total of 14,541 pregnancies were initially enrolled, resulting in 14,676 fetuses. From these pregnancies, there were 14,062 live births, and at 1 year of age, 13,988 children were still alive.^22^, ^23^, ^24^

#### The Insulin Resistance Atherosclerosis Family Study (IRASFS)

A comprehensive description of the study design, recruitment process, and phenotyping in the Insulin Resistance Atherosclerosis Study Family Study (IRASFS) have been described in detail elsewhere.^25^ In summary, this multicenter study aimed to identify the genetic factors contributing to insulin resistance and adiposity.

#### Study of Latinos (SOL)

The Hispanic Community Health Study/Study of Latinos (SOL) is a prospective cohort study conducted in 4 urban field centers across the United States. These centers were deliberately chosen to ensure diversity in terms of national background and behaviors, including diet. A total of 16,415 individuals, who self-identified as having Hispanic or Latino backgrounds, including South Americans, Central Americans, Mexicans, Puerto Ricans, Cubans, and Dominicans, were recruited and participated in data collection between June 2008 and July 2011.

**Figure 1:**
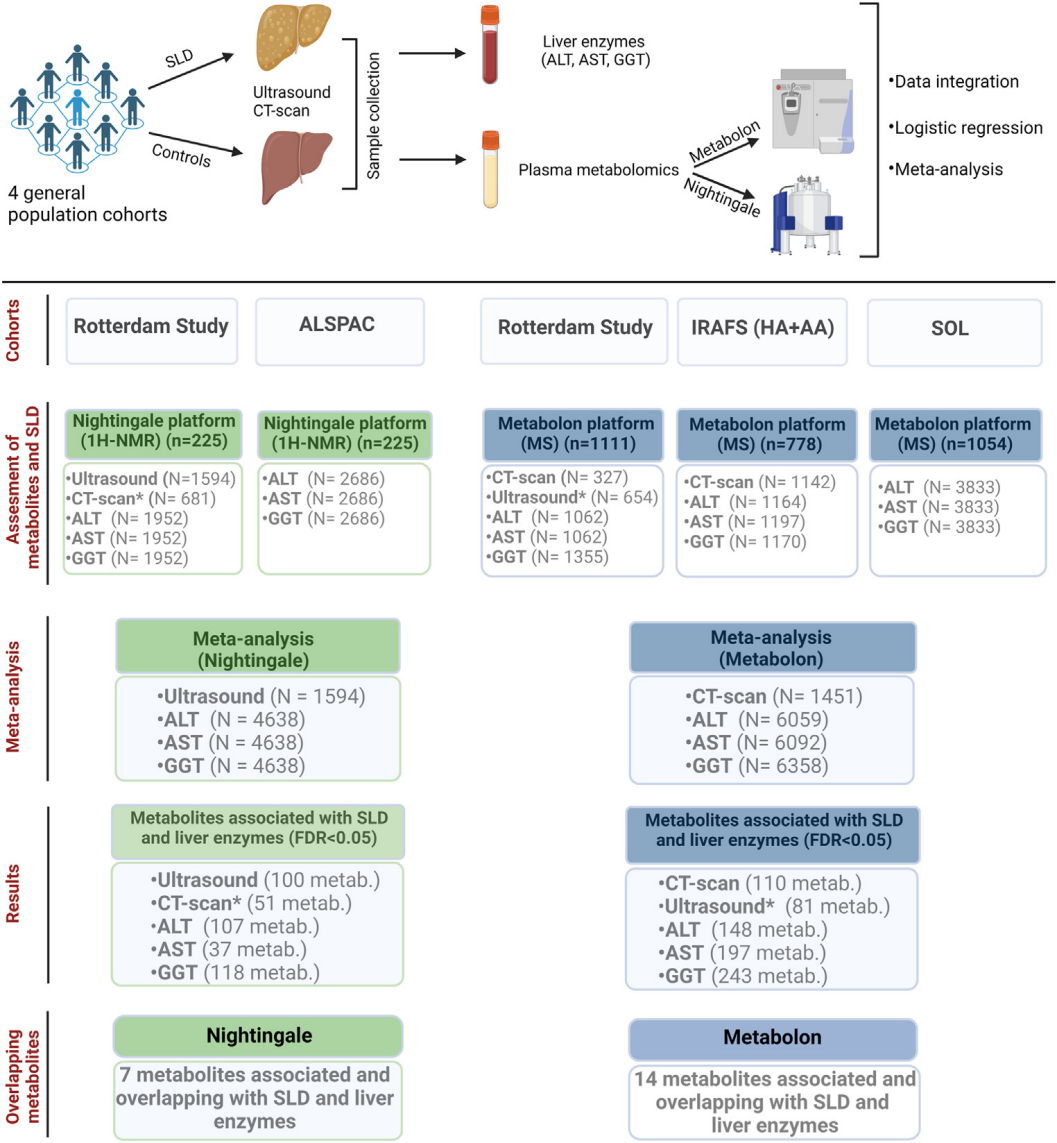
Flowchart of the study participants per cohort. IRASFS (HA + AA), IRASFS (Hispanic American + African American); MS, mass spectrometry; N, sample sizes; metab., metabolites. (∗) indicates independent cohort of RS used only in identifying overlapping metabolites.

A comprehensive overview of the study design alongside all included cohorts, corresponding platforms that have been meta-analyzed together and number of participants are depicted in Figure 1.

### Assessment of Plasma Levels of Metabolites

#### Metabolomics in the RS (Nightingale and Metabolon)

Plasma metabolite levels were measured using the high-throughput 1H–nuclear magnetic resonance Nightingale platform in 1594 participants from 2 separate groups in the RS. These groups, RS-Bios and RS-III-2, were assessed in different years and are independent of each other, with no overlapping participants. The metabolite profiling was part of the Rainbow Project of BioBanking for Medical Research Infrastructure of the Netherlands. The samples were stored at −80 °C for stability. Details on this methodology have been described previously.^26^^,^^27^ A total of 225 plasma metabolites were measured, encompassing lipids, lipoproteins, cholesterol, glycerides, FAs, amino acids, ketone bodies, and inflammation-related compounds. To ensure normal distribution, skewed metabolites were transformed, and measurements were standardized. Missing data were excluded from the statistical analysis. Additional information can be found in the supplementary data with more details. Moreover, plasma levels of metabolites were measured using the (untargeted) Metabolon HD4 platform, containing 1387 metabolites from various pathways, in 1082 RS-I-4 participants. Preprocessing involved excluding 14 participants with excessive missing data and removing metabolites with high missingness and variability. The remaining 1111 metabolites were log2 transformed, and missing data were imputed using the lowest limit of detection. Additionally, 120 metabolites with extensive missingness were removed before imputation, resulting in a dataset of 991 metabolites.

These two high-throughput metabolomics platforms were selected because the Nightingale platform, which uses the nuclear magnetic resonance spectroscopy, allows for the quantification of a targeted set of numerous lipids, lipoproteins, and small molecules in a nondestructive and highly reproducible manner and is particularly an efficient method for large-scale epidemiological studies, while the Metabolon platform uses mass spectrometry to provide a broader and more comprehensive analysis of metabolites, covering a wide range of biochemical pathways, including also some rare and less abundant metabolites that makes it good method for deeper metabolic profiling and discovery research.

#### Metabolomics in replicating cohorts

In the ALSPAC study, over 220 quantified metabolomics measures were obtained per fasted sample of EDTA-plasma/serum (stored at −80 °C), using the Nightingale platform as described previously.^26^^,^^28^ To be comparable with results from the RS, metabolite values were transformed by natural logarithm and scaled to standard deviation units (mean 0, standard deviation 1). In IRASFS, metabolite profiling was performed on stored (at −80 °C) fasting plasma samples collected at the 1999–2002 baseline survey using the untargeted Metabolon platform (Discovery HD4 panel). Similarly, the levels of metabolites were quantified on stored (−70 °C) serum samples using the Metabolon platform in SOL. A comprehensive description of the methodology used to measure the levels of metabolites in each cohort can be found in the supplementary data.

### Assessment of SLD by Abdominal Ultrasound (US) and Computed Tomography (CT) Scan (RS)

Abdominal ultrasonography was conducted using the Hitachi HI VISION 900 machine on all RS participants. Steatotic liver diagnosis followed the criteria set by Hamaguchi et al.,^29^ involving ultrasound (US)-based liver brightness and hepatorenal echo contrast. Secondary causes of steatosis defined as (i) excessive alcohol consumption (>30 g/day for men and >20 g/day for women); (ii) viral hepatitis, based on hepatitis B surface antigen and anti–hepatitis C virus; (iii) use of steatogenic medication, ie, amiodarone, systemic corticosteroids, methotrexate, or tamoxifen and (IV) bariatric surgery, were excluded from all cohorts.

Computed tomography (CT) scans were also performed from February 2002 onwards using 16-slice and 64-slice CT scanners (Siemens). Detailed information regarding the imaging parameters of the scans is provided elsewhere.^30^ Liver fat content was evaluated via liver attenuation from cardiac CT scans. Three circular regions of interest in the liver were measured for mean Hounsfield units (HUs), indicating liver fat content. Liver attenuation values below 40 HU indicated SLD risk. The same CT approach assessed SLD in IRASFS. Additional details are provided in the supplementary data.

### Assessment of Liver Stiffness and MASH (RS)

Liver stiffness measurement was conducted utilizing transient elastography with the FibroScan device (Echosens, Paris, France). The M or XL probe was used to obtain a minimum of 10 measurements. If the interquartile range exceeded 30%, measurements surpassing 7.1 kPa were deemed unreliable and excluded.^31^ Fibrosis was defined as a reliable liver stiffness measurement of ≥8.0 kPa.^32^ Furthermore, an additional sensitivity analysis was performed, using a positive SLD diagnosis based on US findings and elevated serum alanine aminotransferase (ALT) levels ≥ 35 IU/L as an approximation SLD-related steatohepatitis (MASH).^33^

### Assessment of Liver Enzymes

In the RS, blood samples were collected while participants were fasting. Serum aspartate aminotransferase (AST), ALT, and gamma-glutamyl transferase (GGT) levels were determined within 2 weeks after collection using a Merck Diagnostica kit (Merck, Whitehouse Station, NJ, USA) on an Elan Auto-analyzer (Merck). According to local cut-offs, the elevation of GGT was defined as >34 U/L for women and >49 U/L for men. Elevation of ALT was defined as >30 U/L for women and >40 U/L for men, and elevation of AST was defined as >30 U/L for women and >36 U/L for men.^34^ Serum levels of liver enzymes in other cohorts (ALSPAC, IRASFS, and SOL) were measured similarly, more information is provided in the supplementary data.

### Definitions of Covariates

Potential confounders were considered to be any factors known or plausible causal factors for metabolites (or hypothesized exposures) and SLD/liver enzymes (hypothesized outcomes): age, sex, smoking, alcohol, body mass index (BMI), and lipid-lowering medication (LLM).

In all 4 cohorts, information on smoking status (categorized as never, ex-, or current), and alcohol consumption (measured in grams of ethanol per day and categorized as yes/no) were obtained through a questionnaire.^21^ Height and weight of participants were measured while standing up without shoes and heavy outer garments. BMI (kg/m^2^) was calculated as weight divided by squared height in meters. Information regarding the use of LLM was obtained from pharmacy records and home interviews. Diabetes mellitus was defined according to the recent WHO guidelines.^35^ All the covariates included in the statistical analyses were obtained at baseline, at the same time point as the blood collection for metabolites assessment. The definitions of covariates in other cohorts (ALSPAC and SOL) are provided in the supplementary data.

### Statistical Analyses

Per cohort and within the Nightingale platform, the relationship between 225 metabolites and SLD was assessed by multivariable logistic regression models. Natural log-transformed metabolites were used as exposure and the dichotomous SLD diagnosed by US was used as an outcome in the logistic regression models. In model 1, we adjusted for age and sex. In model 2, we additionally adjusted for other confounders, ie for the LLM (yes, no), alcohol consumption (yes, no), BMI (kg/m2), smoking status (current, ex-, or never smokers). To allow comparison between the effect sizes in model 1 and 2, missing values from any covariate were excluded. Sensitivity analyses were conducted by further adjusting for type 2 diabetes in model 3 within the RS subcohorts. The results from the 2 RS subcohorts (RS-III-2 and RS-Bios) were meta-analyzed using fixed-effect models in “METAL” software.^36^

Additionally, we performed multivariable logistic regression to investigate the association between metabolites in the Metabolon platform and SLD diagnosed by CT scan (dichotomous data) and HU measurement (continues data). The number of common metabolites included in the final meta-analysis among the 2 cohorts (RS and IRASFS) was 671.

In RS, a sensitivity analysis was conducted where the outcome variable was replaced with liver stiffness (categorical), MASH, and fibrosis-4, a surrogate marker for liver fibrosis that included age, AST, ALT, and platelets. This was done to examine the relationship between the metabolites and SLD as a spectrum disease.

Multivariable linear regression models were used to examine the association of metabolites in both Nightingale and Metabolon platforms with liver enzymes (GGT, ALT, and AST), using the same model 1 and model 2 adjustments and comparisons as described above (Figure 1).

Lastly, the results of metabolomics analysis with liver enzymes from the 2 RS subcohorts were meta-analyzed with the results from ALSPAC, IRASFS, and SOL cohorts using inverse-variance weighted fixed-effect models. In all pooled results, a false discovery rate corrected *P* < .05 (5%) was used as a significant threshold. The effect of heterogeneity was estimated by *I*^*2*^, which describes the percentage of the total variation in the meta-analysis attributable to study heterogeneity.^37^ All remaining analyses were performed in SPSS statistical software (SPSS, version 25; IBM Crop) and R software version 3.5.2 (The R Foundation for Statistical Computing). The metabolic pathway analysis (MetPA) was conducted using the MetaboAnalyst (V5.0) software (https://www.metaboanalyst.ca/home.xhtml) to check the pathways related to the identified metabolites (Metabolon platform) in both US and CT scan based SLD.

## Results

### Descriptive Characteristics of Cohorts

The baseline characteristics of the study participants in different cohorts are presented in Table 1. Compared to RS, ALSPAC participants were younger and had a higher percentage of current smokers (28.3%). The prevalence of SLD among the RS subcohorts was 29.3% in RS-Bios and 26.0% in RS-III-2. Notably, the RS subcohorts had a higher BMI (27.75 ± 4.2) compared to ALSPAC participants, with a diabetes prevalence of 6.8% among RS participants, likely due to the age differences between the cohorts (62.85, 24.5, 43.37, and 45.82 years for RS, ALSPAC, IRASFS, and SOL, respectively).

**Table 1:** Characteristics of Study Participants

Characteristic	RS-Bios-US^a^			RS-III-2-US^a^			RS-Bios (liver enzymes)	RS-III-2 (liver enzymes)	ALSPAC (liver enzymes)
	All	SLD cases^b^	Controls	All	SLD cases^b^	Controls	All	All	All
Number	663	29.3% (194)	70.7% (469)	931	26.0% (242)	74.0% (689)	545	1476	2687
Age, y	68.31 ± 5.7	68.95 ± 5.6	68.05 ± 5.7	62.06 ± 5.2	62.33 ± 5.2	61.96 ± 5.3	68.39 ± 3.7	62.75 ± 5.8	24.5 ± 0.8
Females, %(N)	58.07 (385)	59.28 (115)	57.87 (270)	58.22 (542)	59.09 (143)	57.91 (399)	58.8 (267)	57.7 (852)	59.1 (1588)
Alcohol consumption, %(N)	86.43 (573)	87.63 (170)	85.93 (403)	87.54 (815)	85.54 (207)	88.24 (608)	85.0 (386)	88.4 (1305)	96.1 (2581)
LLM, %(N)	31.83 (211)	34.54 (67)	30.70 (144)	20.30 (189)	26.45 (64)	18.14 (125)	32.8 (149)	22.1 (326)	NA
Smoking status, % (N)									
Never smokers	33.33 (221)	30.41 (59)	34.54 (162)	35.34 (329)	33.88 (82)	35.85 (247)	31.5 (143)	36.1 (533)	36.5 (981)
Ex-smokers	57.16 (379)	62.37 (121)	55.01 (258)	51.34 (478)	54.55 (132)	50.22 (346)	59.0 (268)	50.2 (741)	35.2 (947)
Current smokers	9.50 (63)	7.22 (14)	10.45 (49)	13.32 (124)	11.57 (28)	13.93 (96)	9.5 (43)	13.7 (202)	28.3 (759)
BMI (kg/m2)	27.75 ± 4.2	30.07 ± 4.0	26.79 ± 3.9	27.37 ± 4.4	30.05 ± 4.6	26.43 ± 4.0	27.76 ± 4.2	27.4 ± 4.5	24.7 ± 4.8
Laboratory data									
AST (U/L)	26.15 ± 10.4	27.43 ± 13.5	25.6 ± 8.7	25.41 ± 14.8	26.09 ± 9.2	25.17 ± 16.3	26.85 ± 12.4	25.7 ± 21.1	27.2 ± 23.4
ALT (U/L)	20.9 ± 11.1	24.1 ± 11.9	19.54 ± 10.5	22.4 ± 14.0	24.7 ± 14.0	21.57 ± 13.9	20.96 ± 12.3	22.6 ± 17.7	25.8 ± 22.7
GGT (U/L)	80.28 ± 64.9	75.57 ± 59.4	82.30 ± 67.3	31.9 ± 34.8	38.2 ± 44.4	29.67 ± 30.1	33.59 ± 30.6	32.6 ± 49.4	20.1 ± 19.6

Characteristic	RS-I-4 (CT scan)^c^			IRASFS-AA (CT scan)^c^			IRASFS-HA (CT scan)^c^			SOL (liver enzymes)
	All	SLD cases^d^	Controls	All	SLD cases^d^	Controls	All	SLD cases^d^	Controls	All
Number	395	24	371	394	18	340	823	128	638	3833
Age, years	62.85 ± 5.2	60.3 ± 4.21	63.01 ± 5.23	43.37 ± 13.54	44.25 ± 12.20	42.99 ± 13.62	41.11 ± 13.38	41.61 ± 11.24	41.05 ± 13.83	45.82 ± 13.80
Females, N (%)	273 (69.1)	16 (66.7)	265 (71.4)	242 (61.42)	13 (72.22)	210 (61.76)	513 (62.33)	67 (52.34)	414 (64.89)	2206 (57.6)
Alcohol consumption, N (%)	344 (87.1)	23 (95.8)	321 (86.5)	216 (54.82)	11 (61.11)	184 (54.12)	442 (53.71)	66 (51.56)	349 (54.70)	1884 (49.2)
LLM, N (%)	53 (13.3)	3 (12.5)	50 (13.5)	29 (7.36)	2 (11.11)	26 (7.65)	33 (4.01)	5 (3.91)	24 (3.76)	450 (11.7)
Smoking status, N (%)										
Never smokers	99 (25.1)	5 (20.8)	94 (25.3)	212 (53.81)	12 (66.67)	186 (54.71)	488 (59.30)	79 (61.72)	382 (59.87)	2252 (58.8)
Ex-smokers	259 (65.6)	16 (66.7)	243 (65.5)	92 (23.35)	3 (16.67)	80 (23.53)	151 (18.35)	25 (19.53)	109 (17.08)	766 (20.0)
Current smokers	37 (9.4)	3 (12.5)	34 (9.2)	90 (22.84)	3 (16.67)	74 (21.76)	184 (22.36)	24 (18.75)	147 (23.04)	815 (21.3)
BMI (kg/m2)	27.46 ± 3.65	30.5 ± 4.13	27.26 ± 3.53	29.86 ± 6.65	33.51 ± 6.37	29.72 ± 6.64	29.12 ± 6.23	32.27 ± 6.02	28.34 ± 5.89	29.78 ± 6.04
Mean liver attenuation (HU)	59.44 ± 9.67	34.1 ± 6.72	61.1 ± 7.23	55.77 ± 8.47	29.01 ± 9.89	57.19 ± 5.54	51.45 ± 12.60	28.71 ± 8.45	56.01 ± 7.20	NA
Laboratory data										
AST (U/L)	NA	NA	NA	18.12 ± 6.98	18.61 ± 4.68	18.18 ± 7.23	19.00 ± 9.39	21.54 ± 10.29	18.53 ± 9.39	24.88 ± 19.71
ALT (U/L)	NA	NA	NA	7.75 ± 5.39	9.22 ± 5.63	7.73 ± 5.43	11.12 ± 10.01	12.88 ± 10.22	10.88 ± 10.19	27.53 ± 24.68
GGT (U/L)	30.92 ± 27.4	32.67 ± 18.03	27.6 ± 16.5	37.40 ± 38.50	35.67 ± 14.50	37.53 ± 40.57	38.28 ± 39.57	46.41 ± 47.40	36.23 ± 38.34	34.92 ± 55.79

a The table shows characteristics of the study participants within the RS with ultrasonography and the study participants in the RS and ALSPAC with liver enzymes data. Values are represented as mean ± (standard deviation), or sample sizes and percentages. RS-Bios consists of RS-I-5, RS-II-3, and RS-III-2, subcohorts of the RS from the same visit with metabolomics data; RS-III-2, an independent Rotterdam subcohort with metabolomics data.

b SLD based on US and liver enzymes per cohort in the metabolomics analysis with the Nightingale platform.

c The table shows characteristics of the study participants within the RS and IRASFS, with CT scan and the study participants in the RS, IRASFS, and SOL with liver enzymes data. Values are represented as mean ± (standard deviation), or sample sizes and percentages. IRASFS-AA, Insulin Resistance Atherosclerosis Family Study–African American; IRASFS-HA, Insulin Resistance Atherosclerosis Family Study–Hispanic American; NA, not applicable.

d SLD-based on CT scan and liver enzymes per cohort in the metabolomics analysis with the Metabolon platform.

In cohorts that used CT scan–based SLD diagnosis, the prevalence of SLD was 6.1% in the RS cohort, 4.5% in IRASFS–African American, and 15.5% in IRASFS–Hispanic American. Additionally, metabolic comorbidities were highly common in the IRASFS subcohorts, as illustrated by the mean BMI of 29.8 ± 6.6 (as shown in Table 1).

### Metabolites Associated with SLD

Through the meta-analysis of the data from the 2 RS subcohorts (RS-Bios and RS-III-2), where SLD was diagnosed by US, we found 100 out of 225 metabolites as well as their ratios in the Nightingale platform to be significantly associated with SLD, after adjusting for multiple testing (false discovery rate < 0.05; Table A1). In model 3, which further adjusts for type 2 diabetes (fully adjusted model), the number of significantly associated metabolites was 108 (Table A2). Additionally, in the first RS subcohort (RS-I-4), where SLD was diagnosed using CT scan, we identified a significant association with 51 metabolites (Tables A3 and A4).

On the Metabolon platform, we found 81 out of 991 metabolites to be significantly associated with SLD diagnosed by US in the RS (Table A5). After adjusting for T2D in the fully adjusted model (model 3), the number of significant metabolites was 94 (Table A6). For SLD diagnosed using CT scan, a meta-analysis combining results from the RS with IRASFS revealed 110 metabolites significantly associated with SLD in dichotomous data (Tables A7 and A8).

### Metabolites Associated with Liver Enzymes

Liver enzymes were used as supplementary information in our analysis. The analysis of metabolites in the Nightingale platform within the RS subcohorts, along with the ALSPAC replicates, and the subsequent meta-analysis of all these cohorts, revealed that various numbers of metabolites are associated with liver enzyme; ALT (N = 107), AST (N = 37), and GGT (N = 118) (Table A9-A11).

In addition, when analyzing the Metabolon results from the RS, IRASFS, and SOL in relation to liver enzymes, a broader range of associations was observed. The meta-analysis across these cohorts revealed that 148 metabolites were significantly associated with ALT, 197 metabolites were associated with AST, and 243 metabolites were associated with GGT (detailed in Table A12-A14).

### Overlapping Metabolites Associated with Both SLD and Liver Enzymes

**Figure 2:**
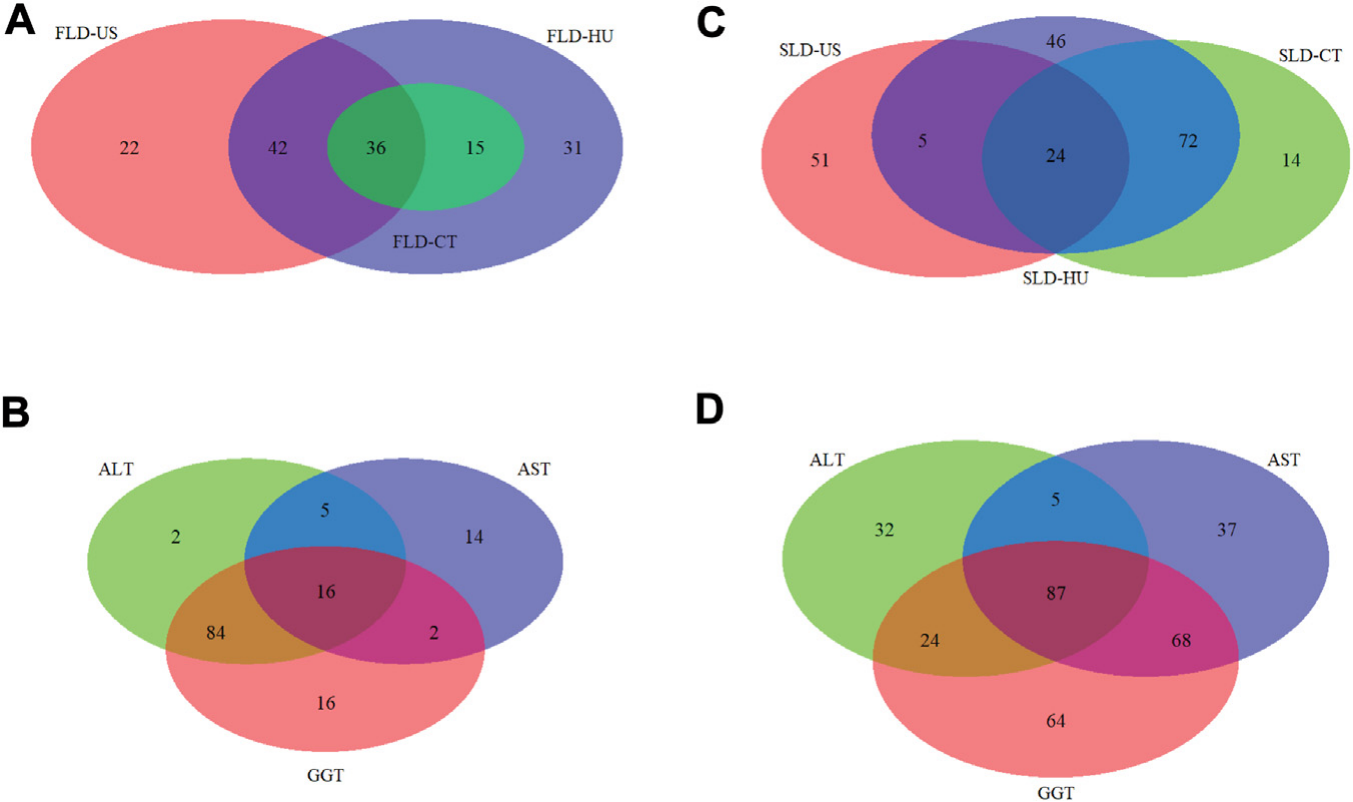
Venn diagram showing the overlapping metabolites assessed by the Nightingale platform in (A) SLD diagnosing utilities, (B) liver enzymes, and by the metabolon platform in (C) SLD diagnosing utilities, (D) liver enzymes. A Venn diagram in (A) showing the number of metabolites (FDR<0.05) that were significantly associated with SLD-US (n = 100), SLD-CT scan (n = 51), and SLD-HU (n = 124) revealed 36 overlapping metabolites whose expression was up/downregulated. While in (B) showing the number of metabolites associated with ALT (n = 107), AST (n = 37), and GGT (n = 118) overlapping revealed 16 metabolites. The diagram in (C) showing the number of metabolites (FDR<0.05) that that were significantly associated with SLD-US (n = 81), SLD-CT scan (n = 110), and SLD-HU (n = 147) revealed 24 overlapping metabolites whose expression was up/downregulated. While in (D) showing the number of metabolites associated with ALT (n = 148), AST (n = 197), and GGT (n = 243) overlapping revealed 87 metabolites. FDR, false discovery rate; GGT, Gamma-glutamyl transferase; n, number of metabolite.

**Figure 3:**
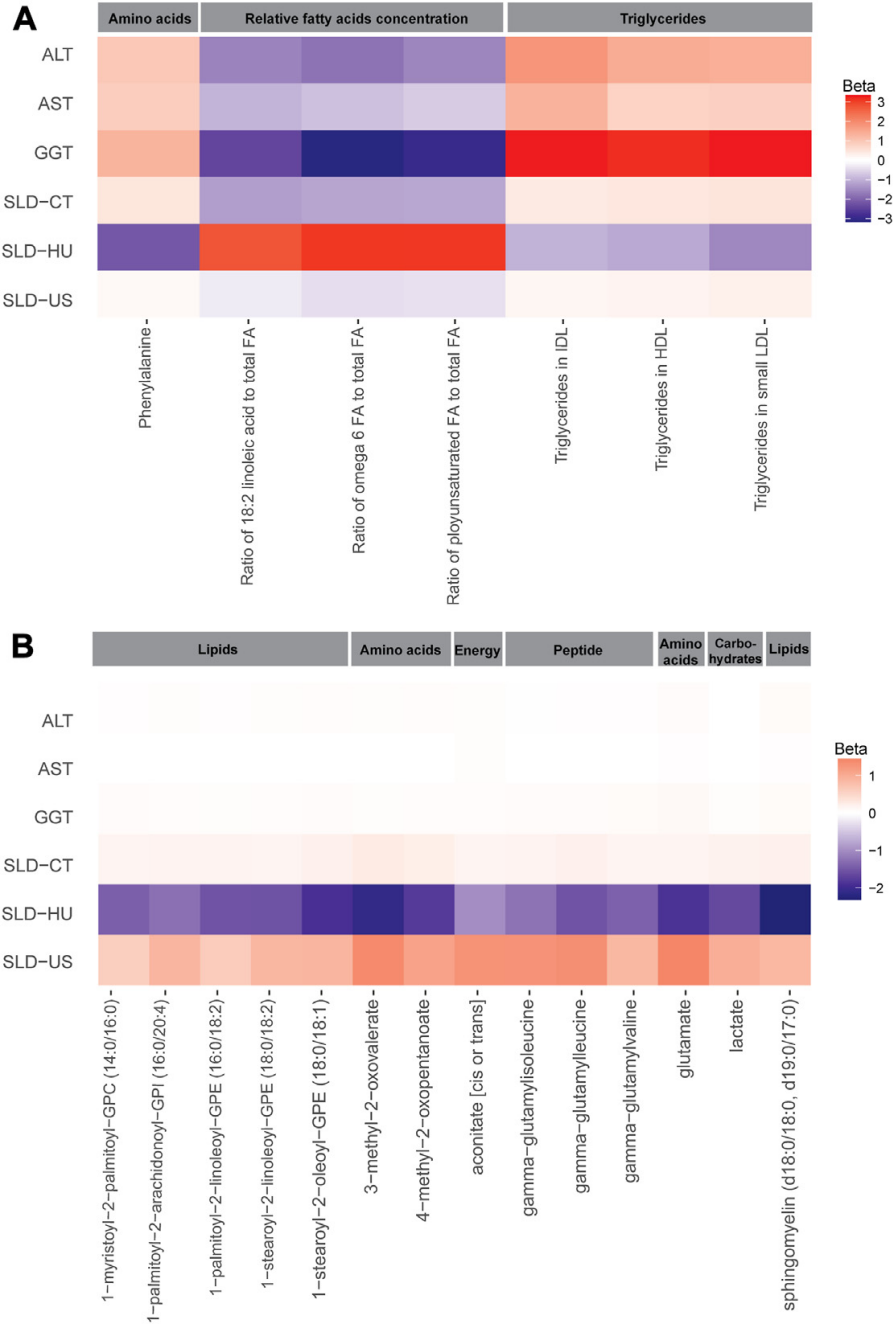
Heatmap visualization of metabolites profiles that were significantly associated and overlapping with SLD based on either US or CT scan and liver enzymes (A) in the Nightingale platform, and (B) Metabolon platform. The color in columns represent the standardized effect estimates (betas). Red color: positive relationship, blue color: negative relationship. The significant threshold is corrected for a FDR adjusted *P* < .05. FDR, false discovery rate; HDL, high-density lipoprotein; IDL, intermediate-density lipoprotein.

The metabolites identified in the Nightingale platform were examined for their association with the prevalence of SLD using US or CT scan, as well as their relationship with liver enzymes (ALT, AST, and GGT). Through this analysis, we aimed to identify any overlapping metabolites among these different measures of liver function and disease. The comparison revealed 36 overlapping metabolites that were associated with SLD diagnosed by both US and CT scan, as displayed in Figure 2A. Additionally, there were 16 overlapping metabolites observed among the different liver enzymes, as presented in Figure 2B. Considering all the different outcomes, including SLD based on US and CT scan, ALT, AST, and GGT, a total of 7 metabolites were found to be overlapping in all traits. These 7 metabolites, namely phenylalanine, triglycerides in high-density lipoprotein, triglycerides in small low-density lipoprotein (LDL), triglycerides in intermediate-density lipoprotein (positive association with SLD), ratio of 18:2 linoleic acids to total fatty acid (FA), ratio of omega 6 FA to total FA, and ratio of polyunsaturated FA to total FA (negative association with SLD) are shown in Figure 3A and Table 2.

**Table 2:** Seven Metabolites From the Nightingale Platform Significantly Associated With Both SLD and Liver Enzymes

Metabolites	ALT		AST		GGT		SLD-HU		SLD-CT		SLD-US	
	Beta	P value	Beta	P value	Beta	P value	Beta	P value	Beta	P value	Beta	P value
Phenylalanine	0.954	1.45E-03	0.868	5.16E-03	1.303	1.91E-04	−2.192	6.78E-09	0.460443	1.13E-02	0.156	2.27E-02
Triglycerides in HDL	1.450	1.73E-06	0.791	9.50E-03	3.188	1.30E-20	−1.104	3.54E-03	0.432096	1.05E-02	0.250	1.09E-04
Triglycerides in small LDL	1.386	5.62E-06	0.816	7.90E-03	3.347	1.73E-22	−1.519	1.07E-04	0.471733	5.19E-03	0.290	5.59E-06
Triglycerides in IDL	1.824	7.08E-09	1.322	1.65E-05	3.325	6.16E-22	−0.985	9.33E-03	0.392824	2.47E-02	0.196	2.18E-03
Ratio of 18:2 linoleic acid to total FA	−1.582	1.35E-06	−0.970	2.90E-03	−2.439	2.50E-12	2.780	1.81E-12	−1.22635	3.76E-06	−0.286	5.36E-05
Ratio of omega 6 FA to total FA	−1.822	1.69E-08	−0.809	1.20E-02	−3.181	7.22E-20	3.103	4.84E-18	−1.1617	1.57E-06	−0.431	5.78E-10
Ratio of polyunsaturated FA to total FA	−1.553	1.42E-06	−0.659	4.06E-02	−2.996	1.02E-17	3.096	3.79E-16	−1.14286	9.28E-07	−0.409	2.69E-09

The table shows 7 metabolites assessed by the Nightingale platform that were significantly associated and overlapped with all studied traits (incl. SLD, diagnosed by US and CT scan and 3 liver enzymes). SLD-US shows the combined results of the 2 RS subcohorts (RS-Bios and RS-III-2). The results for liver enzymes are after meta-analysis of RS-III-2, RS-Bios, RS-1-4, and ALSPAC cohorts. The table shows the results of model 2: adjusted for age, sex, LLM, alcohol consumption, BMI, and smoking status. The significant *P* value set at < 0.05 (FDR).

HDL, high-density lipoprotein; IDL, intermediate-density lipoprotein; FDR, false discovery rate.

Similarly, we followed the same procedure for the Metabolon platform. In this case, we found 24 common metabolites that were associated with both US and CT scan, as depicted in Figure 2C. Additionally, there were 87 metabolites that overlapped among the 3 liver enzymes, as shown in Figure 2D. Overall, there were 14 overlapping metabolites across all the different outcomes (SLD based on US and CT scan, ALT, AST, and GGT), as illustrated in Figure 3B and Table 3. These metabolites mainly belong to the metabolism of lipids, peptides, amino acids, energy, and carbohydrates.

**Table 3:** Fourteen Metabolites From the Metabolon Platform Significantly Associated With Both SLD and Liver Enzymes

Metabolites	ALT		AST		GGT		SLD-CT		SLD-HU		SLD-US	
	Beta	P value	Beta	P value	Beta	P value	Beta	P value	Beta	P value	Beta	P value
1-Myristoyl-2-palmitoyl-GPC (14:0/16:0)	0.034	3.01E-06	0.019	6.32E-06	0.056	6.11E-16	0.149	9.14E-03	−1.441	2.17E-05	0.596	4.76E-02
1-Palmitoyl-2-arachidonoyl-GPI (16:0/20:4)	0.042	2.04E-08	0.019	8.12E-06	0.053	2.62E-14	0.173	1.58E-03	−1.273	1.88E-04	0.879	4.48E-03
1-Palmitoyl-2-linoleoyl-GPE (16:0/18:2)	0.031	6.30E-05	0.011	1.37E-02	0.044	1.26E-09	0.168	4.21E-03	−1.549	5.40E-06	0.633	2.46E-02
1-Stearoyl-2-linoleoyl-GPE (18:0/18:2)	0.042	1.47E-08	0.012	5.81E-03	0.055	2.62E-14	0.159	5.03E-03	−1.571	4.92E-06	0.868	2.55E-03
1-Stearoyl-2-oleoyl-GPE (18:0/18:1)	0.051	9.05E-12	0.016	5.07E-04	0.066	6.00E-20	0.196	8.44E-04	−1.925	1.94E-08	0.896	8.28E-04
3-Methyl-2-oxovalerate	0.041	6.25E-06	0.012	3.35E-02	0.047	5.96E-08	0.269	9.64E-05	−2.142	8.84E-08	1.399	4.35E-03
4-Methyl-2-oxopentanoate	0.050	2.28E-08	0.014	9.34E-03	0.050	7.99E-09	0.231	8.29E-04	−1.813	4.25E-06	1.111	2.85E-02
Aconitate [cis or trans]	0.042	7.83E-08	0.041	3.52E-21	0.050	3.16E-11	0.143	1.76E-02	−1.007	1.47E-02	1.305	1.08E-02
Gamma-glutamylisoleucine	0.028	2.38E-03	0.013	1.09E-02	0.059	1.19E-12	0.166	1.19E-02	−1.259	2.67E-03	1.294	2.60E-03
Gamma-glutamylleucine	0.033	4.07E-04	0.016	3.33E-03	0.058	3.05E-11	0.205	2.21E-03	−1.561	2.43E-04	1.330	1.37E-02
Gamma-glutamylvaline	0.037	1.30E-05	0.020	4.80E-05	0.071	8.09E-20	0.155	1.19E-02	−1.431	1.44E-04	0.857	3.10E-02
Glutamate	0.059	4.28E-13	0.032	1.17E-12	0.101	1.37E-44	0.160	1.15E-02	−1.893	2.47E-07	1.438	3.27E-05
Lactate	0.022	8.36E-03	0.014	2.35E-03	0.045	4.06E-10	0.188	9.97E-04	−1.660	1.84E-06	0.970	3.52E-03
Sphingomyelin (d18:0/18:0, d19:0/17:0)	0.070	4.38E-19	0.033	4.55E-13	0.078	9.03E-24	0.198	9.87E-04	−2.323	2.34E-10	0.846	2.52E-03

The table shows 14 metabolites assessed by the Metabolon platform that were significantly associated and overlapped among all the studied traits (incl. SLD, diagnosed by US and CT scan and 3 liver enzymes). The results of SLD, diagnosed by CT scan and liver enzymes are after meta-analysis of metabolomics data from the RS, IRASFS-AA, IRASFS-HA, and SOL cohorts. The results are based on model 2: adjusted for age, sex, LLM, alcohol consumption, BMI, and smoking status. The significant *P* value set at < 0.05 (FDR).

FDR, false discovery rate.

### Metabolites Associated with Liver Fibrosis and MASH

In our sensitivity analysis within the RS, we observed that among the 255 metabolites assessed using the Nightingale platform, 53 metabolites were nominally associated with liver fibrosis (defined as ≥ 8.0 kPa on a categorical scale) (Table A15). Using the Metabolon platform, we found 40 metabolites associated with liver fibrosis (Table A16). For fibrosis-4, 18 metabolites were significantly associated within the Nightingale platform, and 99 metabolites were significant within the Metabolon platform (Table A17 and A18). Additionally, 103 metabolites were associated with MASH. We further investigated the overlap between the metabolites associated with SLD diagnosed by US and those associated with both liver fibrosis and MASH within Nightingale platform. Our findings revealed that 40 metabolites were common among all 3 conditions (Table A19).

Similarly, we explored the association between metabolites measured using the Metabolon platform and both liver fibrosis and MASH. Our analysis revealed that out of the 991 metabolites examined in the RS, 118 were associated with MASH, while 40 metabolites showed an association with liver fibrosis. Among these, 3 metabolites were found to overlap between SLD based on US and MASH, while 2 metabolites overlapped with liver fibrosis. Additionally, 3 metabolites were jointly linked to both MASH and liver fibrosis (Table A20). These metabolites, involved in lipid, nucleotide, and amino acid pathways.

#### MetPA

**Figure 4:**
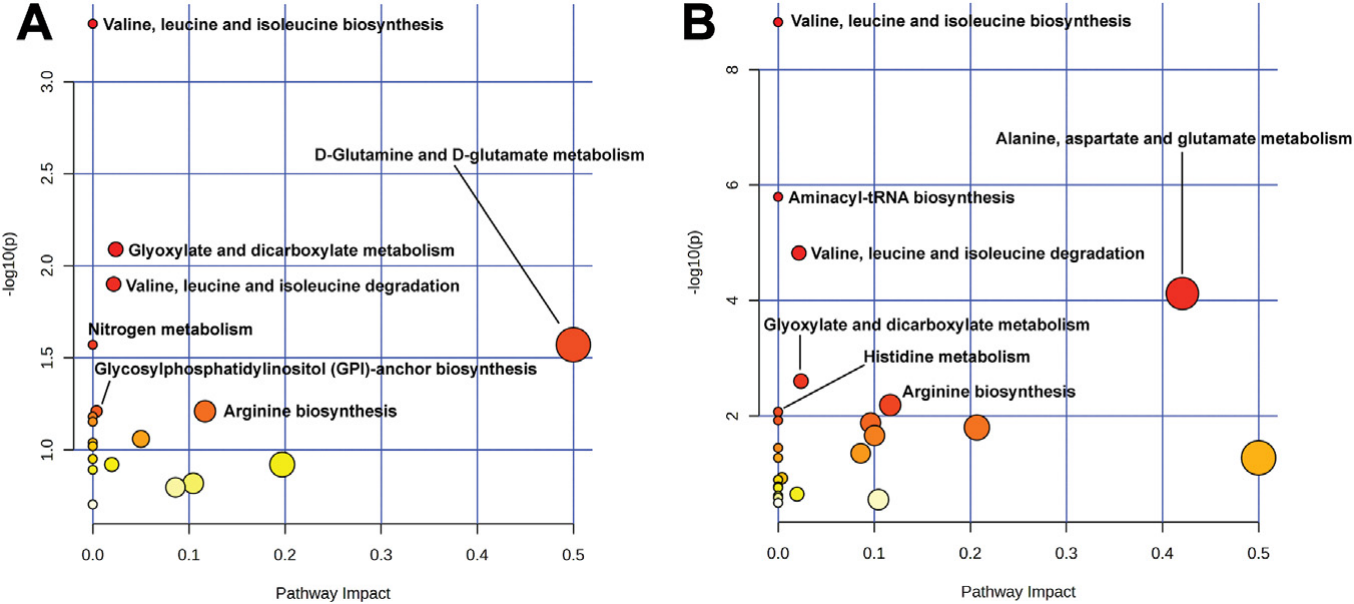
Pathway analysis of the identified metabolites in the Metabolon platform using the over-representation method. (A) Metabolites associated with SLD-based CT scan and US (N = 24) and (B) Metabolites associated with all traits including SLD-based CT scan and US as well as liver enzymes (N = 14). In these metabolome figures, each circle represents a pathway; circle size and color shade are based on the pathway impact and *P* value, respectively (red circles indicates statistical significance and yellow circles indicates not statistical significance). The Human Kyoto Encyclopedia of Genes and Genomes pathway library was used.

We conducted MetPA based on the identified overlapping metabolites. First in SLD diagnosed by US and CT scan (N = 24) and secondly in SLD diagnosed by US and CT scan, and liver enzymes (N = 14) as shown in Figure 4A and B. The identified metabolites were significantly enriched in specific metabolic pathways, including biosynthesis and degradation of the amino acids valine, leucine, and isoleucine. Another important pathway was related to metabolic reactions involved in the synthesis, utilization and/or degradation of glyoxylate and dicarboxylate. Finally, we found D-Glutamine and D-glutamate metabolism, glycosylphosphatidylinositol (GPI)-anchor biosynthesis, and arginine biosynthesis (Figure 4B). While for common metabolites that were associated and overlapping with SLD based on US and CT scan, we found the identified metabolites to be enriched in metabolic pathways such as aminacyl-tRNA biosynthesis, alanine, aspartate, glutamate, and histidine metabolism, in addition to the biosynthesis and degradation of the amino acids valine, leucine and isoleucine, arginine biosynthesis, and histidine metabolism (Figure 4A).

## Discussion

This population-based study explored the association between a wide array of plasma circulating metabolites evaluated through 2 established platforms (Nightingale and Metabolon), with SLD and liver enzymes across 4 cohorts. We found a range of circulating metabolites deriving from lipids, FAs, and amino acids, that could reflect the presence of SLD independently of other metabolic risk factors. Subsequently, we compared the identified metabolites with those associated with liver enzymes, aiming to identify the most important metabolites implicated in liver steatosis pathogenesis. Within discussion of our findings, we highlight 8 out of the 21 overlapping metabolites associated with all the studied traits, while the remaining 13 metabolites—mainly involved in lipid, amino acid, and carbohydrate metabolism—are briefly discussed in Table A21.

Metabolomics studies, especially within the field of hepatology, represent an important and rapidly evolving area of research which has the potential to disclose new insights into the pathophysiology of SLD. By analyzing metabolic profiles, it becomes possible to identify metabolic dysfunctions that precede the diagnosis of SLD. This notion finds support in a significant population-based study conducted in Finland^38^ that the researchers through a longitudinal study noted similar alterations in plasma metabolic profiles up to a decade before the identification of fatty liver. Moreover, within the same study, cross-sectional analysis revealed correlations among a multitude of metabolites and SLD, including notable associations with triglyceride particles within small LDL. Importantly, our research outcomes align closely with the findings of this study. Previous studies have also shown the association of several metabolites, including triglycerides, with SLD and suggested their potential to be used as potential biomarkers for the disease, but few of them have been validated.^18^^,^^39^ Dysregulated lipid metabolism in SLD leads to the formation of small, dense LDL particles characterized by increased triglycerides.^40^ These altered LDL particles are more prone to oxidative modification and glycation, eventually promoting inflammation and endothelial dysfunction, which are key factors in cardiovascular disease development. Moreover, a study found that triglycerides is the strongest predictor for SLD, as compared to other markers of metabolic dysfunction such as high-density lipoprotein, LDL, and serum glucose.^41^ Likewise, previous study has shown that LDL particles are associated positively with both fatty liver and liver enzymes.^42^

On the other hand, and in line with our results, emerging evidence suggests that specific FAs, particularly omega 6 FAs (=n–6 polyunsaturated FAs) and linoleic acid, may play a protective role in the development of SLD.^43^ This observation is reinforced by another study which showed that individuals with steatosis exhibited lower blood levels of omega 6 and omega 3 FAs than those with normal liver function. Finally, it’s noteworthy that omega 6 FAs demonstrated stronger inverse associations with SLD compared to omega 3 FAs.^38^^,^^44^ This evidence provides an insight on the distribution of FAs in the onset of SLD.

Compared to the commonly used Nightingale metabolomics platform, diverse metabolites derived from the Metabolon platform have been studied to a lesser extent in the context of SLD. Here, we found a multitude of metabolites from various metabolic pathways within the Metabolon platform that exhibit significant associations with both SLD and liver enzymes. These metabolites are primarily involved in lipids, amino acids, peptides, and carbohydrate metabolism. Similar to our findings, another study identified several metabolites, including 3-methyl-2-oxovalerate, that showed a positive correlation with liver fat based on proton density fat fraction.^45^ Additionally, other studies have demonstrated that alterations in amino acid concentrations, such as gamma-glutamyl leucine and glutamate, are positively linked with SLD.^46^, ^47^, ^48^ Our analysis also revealed the positive associations of glycolysis-related metabolites such as lactate which previously reflected the presence of SLD.^42^ This finding was supported by a previous study, which established higher lactate and triglyceride levels as associated with hepatocellular carcinoma in cirrhotic livers.^49^ Explicit evidence shows that sphingolipid metabolism is altered in the course of SLD and these changes might contribute to SLD progression.^50^ A previous study reviewed that some sphingolipid species, such as ceramides, may have the potential as biomarkers for SLD.^51^ Altogether, these findings illustrate how multiple pathways in systemic metabolism are troubled before the development of SLD. This insight may aid the prevention or progression of SLD to its severe stages.

Additionally, we identified 3 metabolites from the Metabolon platform (1-linoleoyl-2-arachidonoyl-GPC, orotidine, and p-cresol glucuronide) that are jointly linked to both MASH and liver fibrosis. These metabolites, which are involved in lipid, nucleotide, and amino acid pathways, underscore their potential significance in understanding the progression and management of MASH-related fibrosis. This highlights the need for future investigations to confirm their roles in either promoting or protecting against this progression.

Finally, our pathway analysis showed connections between various identified metabolites and multiple pathways. Predominantly, these pathways are centered around amino acid metabolism, particularly valine, leucine, and isoleucine biosynthesis and degradation, as well as alanine, aspartate, and glutamate metabolism. Another major pathway identified in our study is that of the lipid metabolism. Specifically metabolism of glyoxylate, dicarboxylate, and GPI-anchor biosynthesis, are also associated with SLD irrespective of the extent of obesity and insulin resistance.

Elevated plasma levels of branched-chain amino acids, including valine, leucine, and isoleucine as confirmed in our study, have been previously linked to intrahepatic fat accumulation. These findings suggest a potential connection between hepatic insulin resistance and SLD.^52^ Previous studies have noted that increased plasma amino acid concentrations are most commonly observed in individuals with obesity and SLD, possibly due to heightened insulin resistance and protein catabolism.^46^ Moreover, the genome-scale metabolic modeling of the human gut microbiome has highlighted alterations in glyoxylate and dicarboxylate metabolism within metabolic disorders.^53^ Similarly, pathways associated with lipids, including GPI, have been found to exhibit associations with changes in metabolites related to SLD.^54^

Our study has several strengths. First and foremost, it draws upon relatively large sample size derived from different population-based cohort studies (RS, ALSPAC, IRASFS, and SOL). Second, the study was conducted through the quantification of a vast number of metabolites utilizing 2 widely used metabolomics platforms: Nightingale and Metabolon, known for generating nondestructive, fast, and highly reproducible outcomes.^55^ Third, we incorporated liver enzyme parameters as supportive data alongside SLD data in our investigation, aiming to provide supplementary evidence for the observed associations. Nonetheless, this study has some limitations that must be considered. First, SLD based–US was highly prevalent (26%) within the RS. Due to the unavailability of additional cohorts for replication, our focus was solely directed towards conducting a meta-analysis using the 2 subcohorts of the RS. Second, the included cohorts are not entirely comparable. The difference in age between cohorts particularly ALSPAC cohort might cause distortion within the metabolite profiles. Third, both US’s and CT scans have limitations, with US’s lacking sensitivity in detecting mild steatosis and CT scans exposing patients to radiation.^56^ Future studies should explore more accurate, noninvasive methods such as magnetic resonance imaging or advanced biomarker analysis for improved detection of mild steatosis. Finally, in a cross-sectional observational study, the ability to assess causality is limited. Therefore, future studies warrant to assess the relationship between plasma levels of identified metabolites and the risk of SLD development longitudinally by incorporating additional independent cohorts, preferably from different ethnic groups.

In conclusion, this population-based study demonstrates that plasma levels of several metabolites are significantly associated with liver function and disease. Our findings may help to elucidate metabolic pathways involved in SLD pathogenesis, and the identified metabolites might be considered as potential biomarkers for early diagnosis of the disease. Future studies are yet to be conducted to further validate our findings and more importantly, with a longitudinal design to elucidate whether metabolites may be used as diagnostic or prognostic biomarkers for SLD.
