# Supplementary material for: Plasma Circulating Metabolites Associated With Steatotic Liver Disease and Liver Enzymes: A Multiplatform Population-Based Study
Source: Gastro Hep Adv. 2024 Sep 12;4(2):100551. doi: 10.1016/j.gastha.2024.09.006 (PMC11772964; doi:10.1016/j.gastha.2024.09.006)
Supplement: Supplementary data information [file mmc1.pdf]

Circulating metabolites associated with steatotic liver disease and liver enzymes: a multi-platform population-based study

Yasir J. Abozaid, Ibrahim Ayada, Laurens A. van Kleef, Neil J Goulding, Jessica S. Williams-Nguyen, Robert C Kaplan, Robert J. de Knegt, Lynne E. Wagenknecht, Nicholette D. Allred, Nicholas J Timpson, Jill M. Norris, Yii-Der Ida Chen, M. Arfan Ikram, Willem Pieter Brouwer, Mohsen Ghanbari

Supplementary data information

1.1. Study population

1.1.1 Rotterdam Study (RS)

This study was conducted as part of the Rotterdam Study (RS), a prospective cohort investigation involving individuals aged 45 years and older living in the Ommoord district of Rotterdam, the Netherlands. The RS aims and design have been outlined elsewhere. The initial cohort, known as RS-I, consisted of 7983 individuals aged 55 years or above, established in 1989. A subsequent cohort, RS-II, was introduced in 2000, incorporating an additional 3011 participants who either reached 55 years of age or relocated to the study area. An expansion named RS-III was established in 2006, encompassing 3932 participants aged 45 years and older residing in the research vicinity. Follow-up assessments were conducted at regular intervals, approximately every 3-5 years. All study participants provided written consent to partake in the research and access medical information from their healthcare providers. To conduct this analysis, we included three distinct samples from different cohorts within the RS study, where plasma samples were assessed using the Nightingale platform. Sample 1 comprised participants from visit 4 of RS-I, designated as RS-I-4. Sample 2, referred to as RS-Bios in this manuscript, consisted of a combined sample of participants from visit 5 of RS-I (RS-I-5), visit 3 of RS-II (RS-II-3), and visit 2 of RS-III (RS-III-2). Sample 3 represented another independent set of participants specifically from RS-III-2.

1.1.2 Avon Longitudinal Study of Parents and Children (ALSPAC)

Pregnant women resident in Avon, UK with expected dates of delivery between 1st April 1991 and 31st December 1992 were invited to take part in the study (1-3). The initial number of pregnancies enrolled was 14,541. Of the initial pregnancies, there was a total of 14,676 foetuses, resulting in 14,062 live births and 13,988 children who were alive at 1 year of age. When the oldest children were approximately 7 years of age, an attempt was made to bolster the initial sample with eligible cases who had failed to join the study originally. The total sample size for analyses using any data collected after the age of seven is therefore 15,447 pregnancies, resulting in 15,658 foetuses. Of these 14,901 children were alive at 1 year of age. A total of 14,833 unique women (G0 mothers) enrolled in ALSPAC as of September 2021. Study data were collected and managed using REDCap electronic data capture tools hosted at the University of Bristol (4). REDCap (Research Electronic Data Capture) is a secure, web-based software platform designed to support data capture for research studies. The study website contains details of all the data that is available through a fully searchable data dictionary and variable search tool: http://www.bristol.ac.uk/alspac/researchers/our-data/. For this project, data was used from N=2896 offspring at age 24 who had both metabolite data (from the Nightingale metabolomics platform) and liver enzyme data available.

1.1.3. The Insulin Resistance Atherosclerosis Family Study (IRASFS)

Study design, recruitment and phenotyping in the Insulin Resistance Atherosclerosis Study Family Study (IRASFS) have been described in detail (5). Briefly, this multicenter study was designed to identify genetic determinants of insulin resistance and adiposity. The design of the IRASFS have been described in the supplementary data. The family-based sample includes individuals of self-reported Hispanic American ethnicity from San Antonio, TX, and San Luis Valley, CA, and individuals of self-reported African American ethnicity from Los Angeles, CA. Two clinical examinations were conducted at a 5-year interval. The examinations included an interview for health behaviors and medical history, anthropometric measurements, resting blood pressure and blood drawing for plasma and DNA. Usual consumption of beer, wine and liquor in the past year was assessed by self-report. Exclusions

were made for usual alcohol consumption, which exceeded two drinks/day in men and one drink/day in women. History of liver disease was not collected.

1.1.4. Study of Latinos (SOL)

The Hispanic Community Health Study/Study of Latinos (SOL) is a population-based prospective cohort study conducted at 4 urban field centers in the United States, which were selected to provide diversity in national background and behaviors such as diet. SOL subject recruitment and the study design have been described previously (6, 7). In total, 16,415 individuals who self-identified as Hispanic and/or Latino backgrounds (South Americans, Central Americans, Mexicans, Puerto Ricans, Cubans, and Dominicans) were recruited and completed data collection between June 2008 and July 2011. Detailed data were collected including demographics, socioeconomic status, acculturation, health-related behaviors, medical history, blood laboratory testing in the fasting state, and clinical measurements. Metabolomic profiling was conducted using stored serum samples for a random sample presenting approximately one-third of the cohort (N= 3972). Of these, 3833 participants had information on liver enzymes and covariates.

1.2. Assessment of blood levels of metabolites

1.2.1. Metabolites in RS (Nightingale platform)

The metabolites levels in 1594 participants of the Rotterdam Study (RS-Bios and RS-III-2) were quantified using the high-throughput 1H-NMR Nightingale platform (Nightingale Ltd., Helsinki, Finland), after the collection of fasting EDTA plasma samples. The RS-Bios and RS-III-2 cohorts are independent of each other meaning that the participants are not overlapping and the assessment of metabolites was done in different years. The metabolite profiling was done as part of the 4th Rainbow Project of BioBanking for Medical Research Infrastructure of the Netherlands (BBMRI-NL) (https://www.bbmri.nl/omics-metabolomics/). All samples were stored at -80 °C, which ensured biological stability.. In total 225 plasma metabolites were quantified, which belong to lipid fractions, including

various sizes of lipoproteins, cholesterol levels, glycerides, lipids particles, as well as fatty acids, branched-chain amino-acids, glycolysis-related metabolites, ketone bodies, and metabolites related to inflammation and glycolysis. To ensure normal distribution of the metabolites per cohort, all metabolites belonging to the Nightingale platform with skewed distribution were transformed by natural logarithm or rank transformation was applied before the analysis. The metabolite measurements were then scaled to standard deviation units (mean 0, SD 1) to enable the comparison of results for measures with different units. The missing data of all variables were excluded from the statistical analysis.

1.2.2. Metabolites in RS (Metabolon platform)

We profiled the blood metabolites of 1082 participants of RS-I-4 using the untargeted Metabolon HD4 platform. The Metabolon platform includes 1387 metabolites of different biochemical pathways (including lipids, amino acids, xenobiotics, nucleotides, cofactors and vitamins, peptides, carbohydrates, energy-related metabolites, and uncharacterized metabolites). We performed preprocessing of the metabolomics data before the analyses. To this end, we first excluded 14 participants which had missingness greater than 5 times the standard deviation (SD) of the mean missingness in overall participants. Then, we excluded metabolites with missingness 5 times SD of the mean missingness in metabolites, and coefficient of variance (CV) greater than 30% in internal control samples (NIST Standard Reference Material). Subsequently, we log2 transformed the values of remaining metabolites (N = 1111). We imputed the missing data with the lowest limit of detection. Before performing the imputation, we further removed 120 metabolites with missingness greater than 30% in metabolites.

1.2.3. Metabolomics in IRASFS (Metabolon platform)

Metabolite profiling was performed on stored (at -80 °C) fasting plasma samples collected at the 1999–2002 baseline survey. Metabolite detection and quantification was conducted by Metabolon, Inc. (Durham, North Carolina) using untargeted liquid chromatography-mass spectroscopy (MS) (DiscoveryHD4 panel). Samples were prepared using the automated MicroLab STAR system (Hamilton Company, Salt Lake City, UT). A methanol extraction was used to remove protein, dissociate small molecules bound to protein or trapped in the precipitated protein matrix, and to recover chemically diverse metabolites. The resulting extract was divided into five fractions: two for analysis by two separate reverse phase/ultra-performance liquid chromatography-MS/MS methods with positive ion mode electrospray ionization (ESI), one for analysis by reverse phase/ultra-performance liquid chromatography-MS/MS with negative ion mode ESI, one for analysis by hydrophilic interaction liquid chromatography/ultra-performance liquid chromatography-MS/MS with negative ion mode ESI and one sample was reserved for backup. All methods utilized a Waters ACQUITY ultra-performance liquid chromatography (UPLC) and a Thermo Scientific Q-Exactive high resolution/accurate mass spectrometer interfaced with a heated electrospray ionization source and Orbitrap mass analyzer operated at 35,000 mass resolution. Raw data were extracted, peak-identified and quality control processed using Metabolon's hardware and software. Compounds were identified by comparison to library entries of purified standards or recurrent unknown entities. Peaks were quantified using area under the curve. Several types of controls were analyzed in addition to experimental samples: a technical replicate, pooled matrix sample generated from a small volume of each experimental sample; process blanks, extracted water samples; and QC standards, a cocktail of QC standards chosen not to interfere with the measurement of endogenous compounds were spiked into every analyzed sample, allowed instrument performance monitoring and aided chromatographic alignment. This panel identified and provided relative quantification of known chemical compounds among amino acid, carbohydrate, energy, lipid, nucleotide, and peptide super pathways. In addition to individual named biochemicals; super- and sub-pathways were annotated based on a combination of pathway and chemical structure similarities to serve as a guide for interpretation. Prior to return, data were block corrected for a run day, normalized by batch, and volume extracted. Missing data for metabolites were imputed to the minimum value for

the respective metabolite. Each metabolite in original scale was rescaled to set the median equal to one.

1.2.4. Metabolomics in SOL (Metabolon platform)

Fasting serum samples were collected, processed, and stored at −70 °C from the time of collection until metabolic profiling. Metabolites were quantified using an untargeted liquid chromatography-mass spectrometry (MS)-based metabolomic quantification protocol at Metabolon (Durham, NC, USA) using the Discovery HD4 platform. Detailed procedures are described in (8). The platform captures information for a total of 1136 metabolites, including 782 metabolites with known structural identities and 354 unknown metabolites. Metabolites with missing values for 20% or more of the participants were excluded. For the remaining values, missing values were imputed to half of the limit of detection. Metabolite values were tested for skewness and, if significantly skewed, log10 transformed. Outlier metabolite values (>10 standard deviations from the mean) were set to missing.

2.1. Assessment of SLD by abdominal ultrasound and CT scan (RS)

Abdominal ultrasonography was performed by trained technicians on Hitachi HI VISION 900 in all study participants of the Rotterdam Study. The diagnosis of fatty liver was determined by the ultrasound technician according to the protocol by Hamaguchi et al.. In short, the diagnosis of liver steatosis is based on ultrasonographic liver brightness and hepatorenal echo contrast. SLD is diagnosed by the presence of hepatic steatosis and the absence of the following secondary causes of fatty liver: (1) excessive alcohol consumption (>30 g/day for men and >20 g/day for women), (2) presence of viral hepatitis, (3) use of steatogenic agents, and (4) recent bariatric surgery.

Moreover, from February 2002 onwards trained technicians performed CT-scan on the Rotterdam Study participants. Both 16-slice and 64-slice (N=695) multi-detector CT scanner (Somatom Sensation 16 or 64, Siemens, Forchheim, Germany) was used to perform non-enhanced CT scanning. Using a cardiac CT scan, we evaluated the liver fat content using liver attenuation (LA) using a standardized procedure. First, we placed three circular regions of interest (in cm2) in the liver and calculated the mean Hounsfield unit (HU) within these

regions (9). These regions of interest were drawn throughout the imaged liver tissue (including both the left and right liver lobes) and were carefully chosen to include only liver tissue, and no disruptive tissue such as large blood vessels, cysts, or focal lesions. Next, we calculated the mean HU value from these three measurements as a marker of the total liver fat content (9). Lower LA means a higher risk of fatty liver, so we categorized these variables as well using a threshold of 40HU. Each value below 40HU was considered to be positive for SLD while equal to or above the threshold of 40HU was included in the control group. All measurements were done using Philips iSite Enterprise software (Royal Philips Electronics NV 2006) and are described in detail elsewhere (10).

2.2. Assessment of SLD by CT scan (IRASFS)

The assessment of SLD in IRASFS was performed using CT imaging under a standardized protocol and scans were read centrally at the University of Colorado School of Medicine, Department of Radiology, Bio-Imaging Research Laboratory. Participants received a scout view of the abdomen and pelvis followed by three axial images all during suspended respiration. The three 10-mm-thick images were obtained through the L2–L3, L4–L5 and T11–T12 disc spaces. If the T11–T12 image did not include liver and spleen, a fourth image was obtained by using the scout to determine an appropriate intervertebral disc location. Liver and spleen density were then quantified in Hounsfield Units in the entire liver and spleen as visualized in the slice, excluding any visible vasculature (11). The image obtained at the L4–L5 disc space was used for the determination of visceral adipose tissue (VAT) area; bowel fat is excluded from measurement. All CT images were coded for pathology and image quality; poor-quality studies were excluded from analysis.

3.1. Assessment of liver enzymes (IRASFS)

In IRASFS, alanine transaminase (ALT) and aspartate transaminase (AST) were determined by enzymatic colourimetric assays using a Chemistry Analyzer Model ATAC 8000 (Elan Diagnostic Co., Lakewood, NJ, USA) during the follow-up visit from 2005-2006.

3.2. Assessment of liver enzymes (ALSPAC)

Blood samples were collected whilst the ALSPAC 24-year-old offspring were fasting. GGT, ALT and AST were measured using commercially available enzymatic colorimetric assay kits, manufactured and supplied by Roche diagnostics GmbH, Sandhofer Strasse 116, D-68305 Mannheim.

3.3. Assessment of liver enzymes (SOL)

Serum ALT, AST and GGT were measured in the fasting state using a Roche Modular P Chemistry Analyzer (Roche Diagnostics) with an α-ketoglutaratic enzymatic method as described previously (12, 13). The coefficients of variation were 6.0–7.0% for liver enzymes.

4.1. Definitions of covariates (ALSPAC)

In ALSPAC, we used data collected from the clinic at age 24. Participants were asked for the frequency they had a drink containing alcohol in the past year. We recoded ‘never’ or ‘never had a whole drink’ as no and ‘monthly or less’/’2-4 times a month’/’2-3 times a week’/’>4 times a week’ as yes. The three smoking categories of never/ex/current were derived from questions asking if they had ever smoked a cigarettes and whether they had smoked a cigarette in the last 30 days.

4.2. Definitions of covariates (SOL)

In SOL, alcohol use was derived from participant self-report of current alcohol use (question: “Do you presently drink alcoholic beverages?”). In SOL, participants were instructed to bring containers of those medications taken in the prior month. Medications were inventoried and

therapeutically classified as described previously (14). Participants were classified as taking a lipid-lowering medication if any medication in their individual inventory was coded as “39xxxx” (antihyperlipidemics) or “409925” (calcium channel blocker & HMG CoA reductase inhibitor combined).

Data Availability Statement

Data availability (ALSPAC)

Underlying data

ALSPAC data access is through a system of managed open access. The steps below highlight how to apply for access to the data included in this data note and all other ALSPAC data. The datasets presented in this article are linked to ALSPAC project number B4362, please quote this project number during your application. The ALSPAC variable codes highlighted in the dataset descriptions can be used to specify required variables.

1. Please read the ALSPAC access policy (www.bristol.ac.uk/ media-library/sites/alspac/documents/researchers/data-access/ ALSPAC_Access_Policy.pdf) which describes the process of accessing the data and samples in detail, and outlines the costs associated with doing so.

2. You may also find it useful to browse our fully searchable research proposals database (https://proposals.epi.bristol.ac.uk/ q=proposalSummaries), which lists all research projects that have been approved since April 2011.

3. Please submit your research proposal (https://proposals.epi. bristol.ac.uk/) for consideration by the ALSPAC Executive Com- mittee. You will receive a response within 10 working days to advise you whether your proposal has been approved.

If you have any questions about accessing data, please email alspac-data@bristol.ac.uk.

References

1. Boyd A, Golding J, Macleod J, Lawlor DA, Fraser A, Henderson J, et al. Cohort profile: the ‘children of the 90s’—the index offspring of the Avon Longitudinal Study of Parents and Children. International journal of epidemiology. 2013;42(1):111-27.
2. Fraser A, Macdonald-Wallis C, Tilling K, Boyd A, Golding J, Davey Smith G, et al. Cohort profile: the Avon Longitudinal Study of Parents and Children: ALSPAC mothers cohort. International journal of epidemiology. 2013;42(1):97-110.
3. Northstone K, Lewcock M, Groom A, Boyd A, Macleod J, Timpson N, et al. The Avon Longitudinal Study of Parents and Children (ALSPAC): an update on the enrolled sample of index children in 2019. Wellcome open research. 2019;4.
4. Harris PA, Taylor R, Thielke R, Payne J, Gonzalez N, Conde JG. Research electronic data capture (REDCap)—a metadata-driven methodology and workflow process for providing translational research informatics support. Journal of biomedical informatics. 2009;42(2):377-81.
5. Henkin L, Bergman RN, Bowden DW, Ellsworth DL, Haffner SM, Langefeld CD, et al. Genetic epidemiology of insulin resistance and visceral adiposity: the IRAS Family Study design and methods. Annals of epidemiology. 2003;13(4):211-7.
6. Sorlie PD, Avilés-Santa LM, Wassertheil-Smoller S, Kaplan RC, Daviglus ML, Giachello AL, et al. Design and implementation of the Hispanic Community Health Study/Study of Latinos. Ann Epidemiol. 2010;20(8):629-41.
7. LaVange LM, Kalsbeek WD, Sorlie PD, Avilés-Santa LM, Kaplan RC, Barnhart J, et al. Sample design and cohort selection in the Hispanic Community Health Study/Study of Latinos. Annals of epidemiology. 2010;20(8):642-9.
8. Kaplan RC, Williams-Nguyen JS, Huang Y, Mossavar-Rahmani Y, Yu B, Boerwinkle E, et al. Identification of Dietary Supplements Associated with Blood Metabolites in the Hispanic Community Health Study/Study of Latinos Cohort Study. The Journal of Nutrition. 2023;153(5):1483-92.
9. Kodama Y, Ng C, Wu T-T, Ayers G, Curley S, Abdalla E, et al. Comparison of CT methods for determining the fat content of the liver. 2007.
10. Wolff L, Bos D, Murad SD, Franco OH, Krestin GP, Hofman A, et al. Liver fat is related to cardiovascular risk factors and subclinical vascular disease: the Rotterdam Study. European Journal of Echocardiography. 2016;17(12):1361-7.
11. Davidson LE, Kuk JL, Church TS, Ross R. Protocol for measurement of liver fat by computed tomography. Journal of applied physiology. 2006;100(3):864-8.
12. Kallwitz ER, Daviglus ML, Allison MA, Emory KT, Zhao L, Kuniholm MH, et al. Prevalence of suspected nonalcoholic fatty liver disease in Hispanic/Latino individuals differs by heritage. Clinical Gastroenterology and Hepatology. 2015;13(3):569-76.
13. Thyagarajan B, Howard AG, Durazo-Arvizu R, Eckfeldt JH, Gellman MD, Kim RS, et al. Analytical and biological variability in biomarker measurement in the Hispanic Community Health Study/Study of Latinos. Clinica Chimica Acta. 2016;463:129-37.
14. Faurot KR, Siega-Riz AM, Gardiner P, Rivera JO, Young LA, Poole C, et al. Comparison of a medication inventory and a dietary supplement interview in assessing dietary supplement use in the Hispanic community health study/study of Latinos. Integrative Medicine Insights. 2016;11:IMI. S25587.
